# Restoring the Extracellular Matrix: A Neuroprotective Role for Collagen Mimetic Peptides in Experimental Glaucoma

**DOI:** 10.3389/fphar.2021.764709

**Published:** 2021-11-02

**Authors:** Nolan R. McGrady, Silvia Pasini, Robert O. Baratta, Brian J. Del Buono, Eric Schlumpf, David J. Calkins

**Affiliations:** ^1^ Department of Ophthalmology and Visual Sciences, Vanderbilt Eye Institute, Vanderbilt University Medical Center, Nashville, TN, United States; ^2^ Stuart Therapeutics, Inc., Stuart, FL, United States

**Keywords:** ocular collagen, collagen mimetic peptides, glaucoma, optic neuropathy, collagen reparative, extracellular matrix, neuroprotection

## Abstract

Optic neuropathies are a major cause of visual disabilities worldwide, causing irreversible vision loss through the degeneration of retinal ganglion cell (RGC) axons, which comprise the optic nerve. Chief among these is glaucoma, in which sensitivity to intraocular pressure (IOP) leads to RGC axon dysfunction followed by outright degeneration of the optic projection. Current treatments focus entirely on lowering IOP through topical hypotensive drugs, surgery to facilitate aqueous fluid outflow, or both. Despite this investment in time and resources, many patients continue to lose vision, underscoring the need for new therapeutics that target neurodegeneration directly. One element of progression in glaucoma involves matrix metalloproteinase (MMP) remodeling of the collagen-rich extracellular milieu of RGC axons as they exit the retina through the optic nerve head. Thus, we investigated the ability of collagen mimetic peptides (CMPs) representing various single strand fractions of triple helix human type I collagen to protect RGC axons in an inducible model of glaucoma. First, using dorsal root ganglia maintained *in vitro* on human type I collagen, we found that multiple CMPs significantly promote neurite outgrowth (+35%) compared to vehicle following MMP-induced fragmentation of the *α*1(I) and *α*2(I) chains. We then applied CMP to adult mouse eyes *in vivo* following microbead occlusion to elevate IOP and determined its influence on anterograde axon transport to the superior colliculus, the primary RGC projection target in rodents. In glaucoma models, sensitivity to IOP causes early degradation in axon function, including anterograde transport from retina to central brain targets. We found that CMP treatment rescued anterograde transport following a 3-week +50% elevation in IOP. These results suggest that CMPs generally may represent a novel therapeutic to supplement existing treatments or as a neuroprotective option for patients who do not respond to IOP-lowering regimens.

## Introduction

Optic neuropathies are a major cause of vision loss and disability worldwide. These are collectively characterized as conditions that involve the specific degeneration of retinal ganglion cell (RGC) axons, approximately 1.5 million of which comprise the optic nerve and retinal projection to the visual brain. Glaucoma is the most common chronic optic neuropathy. Progression evolves from sensitivity to intraocular pressure (IOP), the hallmark feature of glaucoma, with age exacerbating risk. Approximately 112 million will be afflicted worldwide by 2040 (Tham et al., 2014), with annual direct costs for IOP-lowering treatments in the United States alone approaching $6 billion (Rein, 2013). Despite this investment, an estimated 40–50% of patients will still progress to irreversible vision loss due to continuing degeneration (Leske et al., 2003; Susanna et al., 2015). Since the optic projection is part of the central nervous system (CNS), it does not regenerate intrinsically (Calkins et al., 2017; Wareham et al., 2020). Thus, there is substantial need for new therapeutics that prevent or treat vision loss by directly promoting survival of RGCs and their axons independent of the etiological roots of degeneration.

Stress from sensitivity to IOP in glaucoma is conveyed at the optic nerve head, where RGC axons pass unmyelinated in exiting the retina to form the myelinated optic nerve proper. From there, RGC degeneration proceeds in both directions. A distal degenerative program includes early decline of anterograde axon transport to central brain targets, followed by disassembly of the myelinated axon in the optic nerve (Crish et al., 2010; Crish and Calkins, 2015). Correspondingly, the proximal degenerative program reduces the RGC dendritic arbor, which loses complexity as excitatory synapses are eliminated in a complement-dependent process (Williams et al., 2016; Risner et al., 2018). Importantly, RGC bodies and the unmyelinated axon segment in the retina persist until much later in progression (see Calkins, 2012), enabling both to adapt early in progression to extend axon signaling as long as possible (Calkins, 2021). This persistence lies at the root of hope for regenerative therapies to restore the distal axon projection and visual signaling to the brain (Calkins et al., 2017).

The optic nerve head is an important site of pathological changes in glaucoma for other reasons as well. In the adult CNS, the extracellular matrix (ECM) plays a critical role as a biologically active scaffold for maintaining biophysical and biomechanical stability and structure and as a mediator for the diffusion and availability of signaling molecules, such as those mediating interactions between axons and astrocytes (Barros et al., 2011). Collagen is highly abundant, and the matrix of the retina and optic nerve contain regions of highly concentrated collagen types I, III, IV, V, and VI (Morrison et al., 1988; Huang et al., 2013). This holds true for the optic nerve head as well, where collagen concentrates densely as part of the extra-axonal milieu (Sawaguchi et al., 1999). There, astrocyte glia cells form a dense lateral plexus that secretes collagen as a key element of the ECM to support RGC axon function (Hernandez, 2000). In glaucoma, disruptions in this collagen are part of remodeling of the matrix, which includes both protease-mediated breakdown and generation or deposition of new matrix (Chintala, 2006; Wallace et al., 2014; Schneider and Fuchshofer, 2016). Similar protease-mediated collagen degradation and remodeling occurs in glaucomatous retina (Gupta et al., 2017; Hernandez et al., 1990), and elevated IOP is associated with increased levels of collagens I, IV and VI in retina and optic nerve head (Hernandez, 2000; Guo et al., 2005; Schneider and Fuchshofer, 2016). In the CNS, as the ECM and its collagen continue to remodel and neural tissue is lost, formation of a glial scar, necessary to maintain nerve tract volume, presents a physical and biochemical barrier to axon repair in disease and injury (Bradbury and Carter, 2011; Soleman et al., 2013).

Thus, in CNS disease and injury, biochemical degradation and remodeling of the ECM is viewed as a critical factor in neurodegeneration (Maguire, 2018). Therapies that rebuild or leverage the matrix hold great therapeutic potential to promote neural repair and regeneration (Ren et al., 2015; Song and Dityatev, 2018). A signature characteristic of collagen is a triple helical structure, in which individual collagen triple helices are knowns as tropocollagen (TC). This structure includes a set of three polypeptide chains comprising repeating sequences of glycine-x-y triplets, where x and y often (but not always) represent proline and hydroxyproline (Kadler et al., 2007). Collagen mimetic peptides (CMPs) show great promise for their capacity to directly repair damaged triple helical collagen. This is especially so for therapies utilizing type I collagen, which is known for its very low antigenicity, robust bioavailability, and capacity to be modified for therapeutic platforms (Hapach et al., 2015). Mimetics of type I show promise in remodeling endogenous collagen damaged in tissues ranging from cartilage to peripheral nerve (Fallas et al., 2010). Fractional single strand CMPs of type I collagen are specifically designed to intercalate into fragmented and partially digested triple helices, thus restoring reforming the native triple helix and is role in maintaining structure and signaling (Chattopadhyay et al., 2012, 2016; Chattopadhyay and Raines, 2014). Recently we demonstrated that a mimetic of type I collagen was efficacious in promoting repair and structural integrity of the corneal epithelium following an acute injury (Baratta RO. et al., 2021). Similarly, fragments of collagens IV, XV and XVIII promote the growth of blood vessels and tumor cells and influence a variety of other cellular activities (Ortega and Werb, 2002).

Given the therapeutic potential of repairing damaged collagen in the ECM and the importance of an intact ECM to CNS structure and function, we tested the capacity of CMPs to promote neurite outgrowth in culture under conditions of collagen fragmentation. We found that multiple CMPs demonstrate reparative capacity *in vitro*, enhancing both length and coverage of outgrowth from dorsal root ganglia explants. To test their therapeutic capacity *in vivo*, we found that ocular delivery of a CMP prevented functional deficits in the RGC projection to the brain following induced elevations of IOP in the most prevalent model of experimental glaucoma, microbead occlusion (Calkins et al., 2018; Calkins, 2021). Thus, CMPs may have broad therapeutic potential to protect neurons in disease or injury under duress from fragmented collagen in the extra-axonal milieu as is commonly seen in disease or injury.

## Materials and Methods

### Collagen Digestion Protein Assay

Following Galazka et al. (1996), we activated 60 nM metalloproteinase 1 (MMP-1, Biolegend, San Diego, CA) with 2 mM 4-aminophenylmercuric acetate (APMA, Sigma, St. Louis, MO) at 37°C in TCN buffer (25 mM Tris-HCl, 10 mM CaCl2, 150 mM NaCl, pH 7.5) for 30 min as an expediency, before adding human type 1 atelocollagen (3.375 mg, 0.2 mg/ml, Advance BioMatrix, Carlsbad, CA), which is a soluble form of tropocollagen with little or no immunogenicity (Hanai et al., 2012). This mixture was incubated at 37°C for an additional 6 h (h). We measured collagen digestion with SDS-PAGE using 4–20% polyacrylamide gels (Bio-Rad Laboratories, Hercules, CA) under reducing conditions; longer incubation periods did not increase levels of digested collagen. We stained the gels with EZ-Run Protein gel staining solution for 60 min followed by multiple washes with double distilled water (DDW) and imaged the results using the Bio-Rad ChemiDoc MP imaging system under ultraviolet (UV) light (n = 9 samples per condition).

### Dorsal Root Ganglia Cultures

Twelve-well culture plates were coated overnight at 37°C either with MMP-1-digested collagen as prepared above or with intact human type 1 atelocollagen, both diluted with DDW to a concentration of 100 mg/ml, followed by multiple washings with DDW to remove unbound substrate. We produced four distinct single-strand, 7-repeat, 21-residue collagen mimetic peptides (CMPs) analogous to CMPs that have been reported to intercalate with high affinity and selectivity into damaged type I collagen *in vitro* and *in vivo* (Chattopadhyay et al., 2012, 2016; Chattopadhyay and Raines, 2014). These were manufactured in limited quantity by Bachem, AG (Germany), with the following sequences: CMP 05A (Pro-Hyp-Gly)_7_, CMP 09C (Pro-trans-Flp-Gly)_6_-Pro-Cys-Gly, CMP 10A (Hyp-Pro-Gly)_7_-P, and CMP 13A (cis-Flp-Hyp-Gly)_7_, where proline (Pro), hydroxyproline (Hyp), glycine (Gly), 4-fluoro-proline (Flp), and the undecapeptide tachykinin neuropeptide substance P (P) are abbreviated as indicated.

After washing with DDW, plates were coated either with CMP (100 μl; 100 µM) or vehicle (DDW) and incubated at 37°C for 5 h. The coating solutions were removed, and the plates washed with DDW. We aseptically dissected and cultured dorsal root ganglia (DRG) from E19 Sprague-Dawley rats (Charles River, Wilmington, MA) onto the pre-coated plates in AN_2_ medium (n = 16–18 explants per condition). This contained Minimum Essential Media (MEM) with 10% calf bovine serum (Thermo Scientific, Waltham, MA), as well as 50 μg/ml ascorbic acid, 1.4 mM L-glutamine, and 5 ng/ml nerve growth factor (Thermo Scientific, Waltham, MA). We added 0.6% glucose and 10^–5^ M fluoro-2′-deoxyuridine (Sigma-Aldrich, St. Louis, MO) and incubated in a 5% CO_2_ humidified incubator at 37°C. After 48 h, we prepared complete montages of each DRG explant in culture. These were collected from multi-focus stacks of high-magnification (100x) photomicrographs using phase-contrast optics and a motorized stage with autofocus set to optimize edge detection of individual neurites. Using Fiji ImageJ software (https://imagej.net/ImageJ), we measured from these montages the area of the field extending from the edge and surrounding the explant with neurite coverage and the longest neurite extending from the edge of each explant. We chose DRG explants for these initial studies since previous work has shown that intact collagen is an important substrate for DRG growth, which is quite robust in culture (Sango et al., 1993; Hari et al., 2004).

### Animal Studies

Recently we found that ocular delivery of a CMP was efficacious in promoting repair of corneal collagen *in vivo* without noticeable side effects to the eye (Baratta RO. et al., 2021). Here we used this same CMP (CMP 03A, (Pro-Pro-Gly)_7_) also manufactured by Bachem, AG (Germany) for use in our glaucoma model; an additional batch was produced for other purposes with attachment of the Tide Fluor™ 2 (AAT Bioquest, Sunnyvale, CA, United States) moiety as a fluorescent reporter. The Vanderbilt University Institutional Animal Care and Use Committee approved all experimental procedures. Following our previous publications (Sappington et al., 2010; Chen et al., 2011; Weitlauf et al., 2014; Calkins et al., 2018; Cooper et al., 2020), we used microbead occlusion to elevate intraocular pressure (IOP) unilaterally in 6 to 8-week-old C57BL/6 mice (n = 5 per cohort; Charles River Laboratory, Wilmington, MA). Briefly, for 1–2 days prior to elevation, we obtained baseline IOP measurements bilaterally in anesthetized (2.5% isoflurane) mice using TonoPen XL (Medtronic Solan, Minneapolis MN United States), which were averaged (day 0). Following these measurements, we elevated IOP unilaterally by injecting 1.5 µl of 15 µm polystyrene microbeads (Invitrogen, Carlsbad CA United States) into the anterior chamber; the fellow eye received an equal volume of sterile saline to serve as an internal control. We measured IOP weekly for the duration of the experiment. Both eyes received vehicle (phosphate buffered saline or PBS) or CMP (in PBS) *via* a single intravitreal injection (1.5 µl) at day 10 post-microbead injection; topical application (10 µl) gave indistinguishable results in pilot studies. During handling, mice were maintained on a 12 h light-dark cycle with standard rodent chow available *ad libitum*.

Two days prior to sacrifice, we anesthetized mice with 2.5% isoflurane and injected 1.5 μl of 1 mg/ml solution of cholera toxin subunit B (CTB) conjugated to Alexa-555 (Molecular Probes, Eugene, OR) into the vitreous body of both eyes following our established protocol for mice (Ward et al., 2014; Calkins et al., 2018; Risner et al., 2021). The mice were perfused transcardially with PBS followed by 4% paraformaldehyde and the brains dissected, and cryoprotected in 30% sucrose. We prepared coronal midbrain sections (50 µm) using a freezing sliding microtome and photographed alternating sections of the superior colliculus using a Nikon Ti Eclipse microscope (Nikon Instruments Inc, Melville, NY). We quantified the intensity of CTB signal (intact transport) using a custom ImagePro macro (Media Cybernetics, Bethesda, MD) as previously described in several of our publications (Weitlauf et al., 2014; Lambert et al., 2019; Cooper et al., 2020; Risner et al., 2021). All animal experiments were approved by the Vanderbilt University Medical Center Institutional Animal Care and Use Committee. We did not observe overt signs of ocular inflammation, irritation, or other pathology associated with intraocular injections or treatment. consistent with our previous studies utilizing multiple injections (e.g., Cooper et al., 2020; Risner et al., 2021).

### Statistical Analysis

All data are presented as mean ± standard error of the mean (SEM). Statistical analyses and graphs were made using Sigma Plot Version 14 (Systat, San Jose, CA). Outlier analysis was performed using Grubbs’ test (Graphpad Software, San Diego CA). Parametric statistics were performed (*t*-test, analysis of variance) if data passed normality and equal variance tests; otherwise, we performed non-parametric statistics (Mann-Whitney, ANOVA on Ranks, Welch’s test). Statistical significance was defined as *p* ≤ 0.05.

## Results

### Collagen Mimetic Peptides Enhance Neuronal Outgrowth In Vitro

**FIGURE 1:**
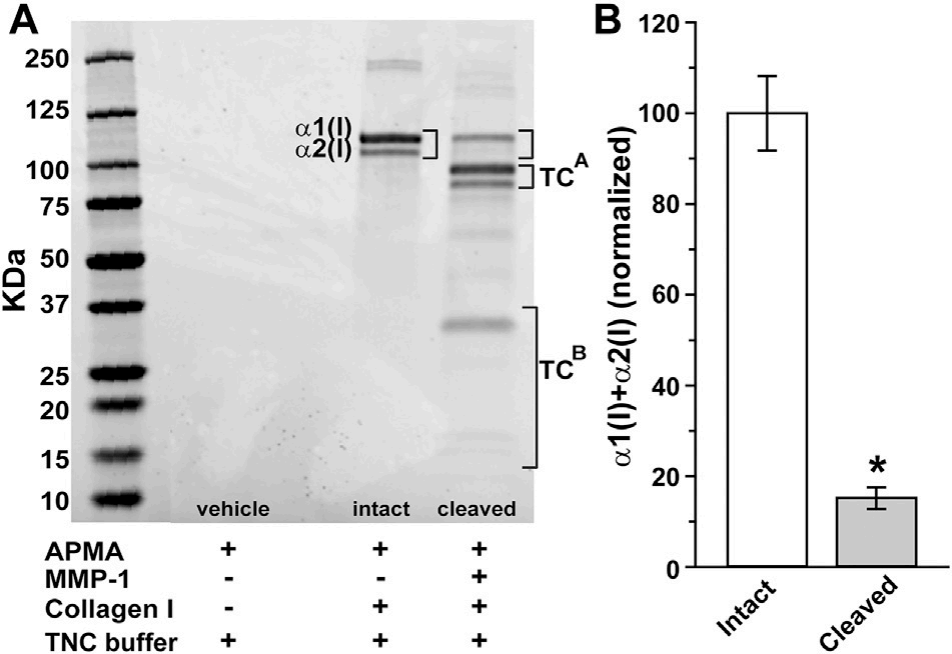
Digestion of collagen I by matrix metalloproteinase 1 (MMP-1). **(A)** SDS-PAGE electrophoresis gel shows dual bands representing reaction products for intact α1(I) and α2(I) chains of human type 1 atelocollagen (brackets). Cleavage with activated MMP-1 (60 nM) reduces both α1(I) and α2(I) while increasing their ¾ (TC^A^) and ¼ (TC^B^) fragments, respectively. **(B)** Level of combined full length of α1(I) and α2(I) chains reduce by 85% following MMP-1 cleavage relative to intact collagen (*, *p* < 0.001; n = 9 for each condition).

SDS-PAGE electrophoresis demonstrated dual bands characteristic of type I collagen corresponding to intact α1(I) and α2(I) chains (Figure 1A). These denatured to their ¾ length (TC^A^) and ¼ length (TC^B^) fragments, respectively, following digestion with MMP-1, corresponding to cleavage roughly ¾ length away from the N terminus (Chung et al., 2004). We found that MMP-1 cleavage reduced the level of combined full length of α1(I) and α2(I) chains by 85% following relative to intact chains (*p* < 0.001; Figure 1B).

**FIGURE 2:**
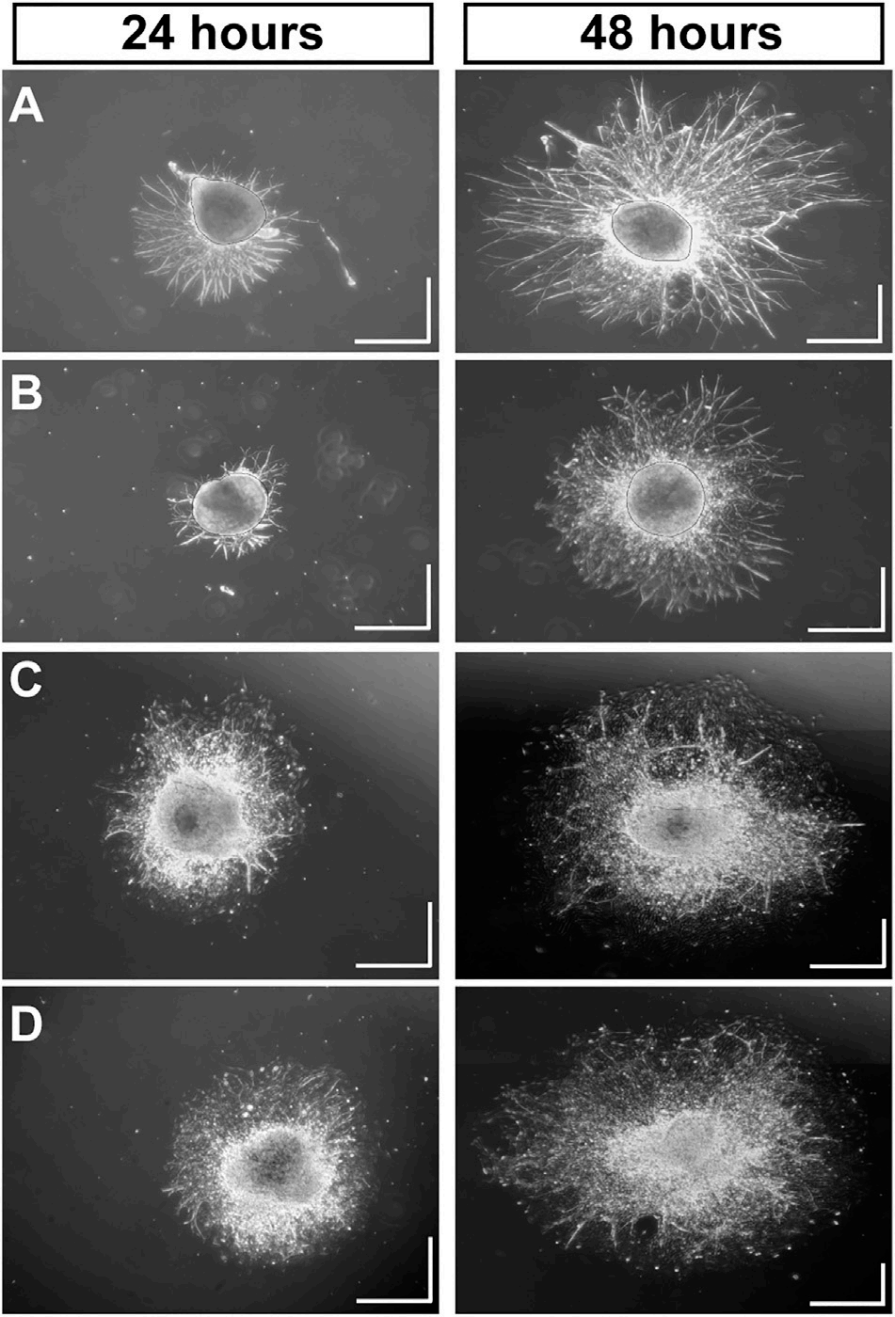
Mimetic Peptides are Neurotrophic. Dorsal root ganglia (DRG) explants 24 and 48 h after plating on intact collagen **(A)** vs. partially digested collagen treated with vehicle **(B)**. Treatment with CMP 10A **(C)** and CMP 09C **(D)**, both at a concentration of 100 μM, increases DRG neurite outgrowth and the area of the field around the explant with neurite coverage compared to vehicle. Scale = 0.5 mm in each direction.

**FIGURE 3:**
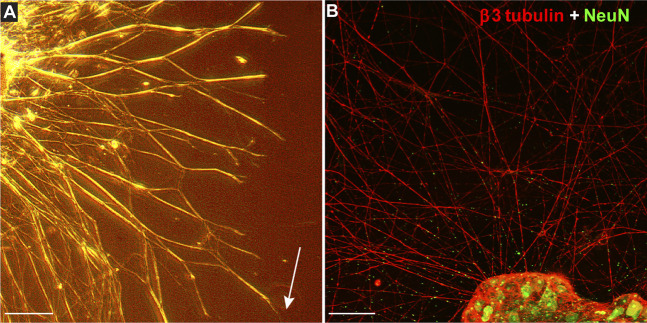
Measuring Explant Outgrowth. **(A)** DRG explant 48 h after plating on intact collagen visualized live through multi-focal high-magnification phase contrast microscopy to optimize identification and measurement of longest neurite (arrow). Confocal micrograph of similar explant **(B)** shows neurites immune-labeled for β3-tubulin (red) extending from DRG neurons labeled against NeuN (green). Scale = 250 **(A)** or 100 **(B)** µm.

Using MMP-1 to cleave type I collagen as shown (Figure 1), we tested how degradation influences growth of neurites from DRG explants and whether CMP treatment counters the effect. After 24 h, montages of collapsed z-stacks of phase contrast micrographs of explants on intact collagen showed modest neurite outgrowth, which increased substantially by 48 h (Figure 2A). Plating on damaged collagen reduced the extent of outgrowth (Figure 2B), while explants plated on damaged collagen treated with two distinct CMPs (each at 100 µM) demonstrated improved outgrowth (Figures 2C,D). To measure neurite outgrowth accurately, we visualized explants live using multi-focal high-magnification phase contrast microscopy to optimize identification and measurement (Figure 3A). This method was preferred to post hoc measurement of neurites visualized post-fixation using antibodies against β3 tubulin (Figure 3B).

**FIGURE 4:**
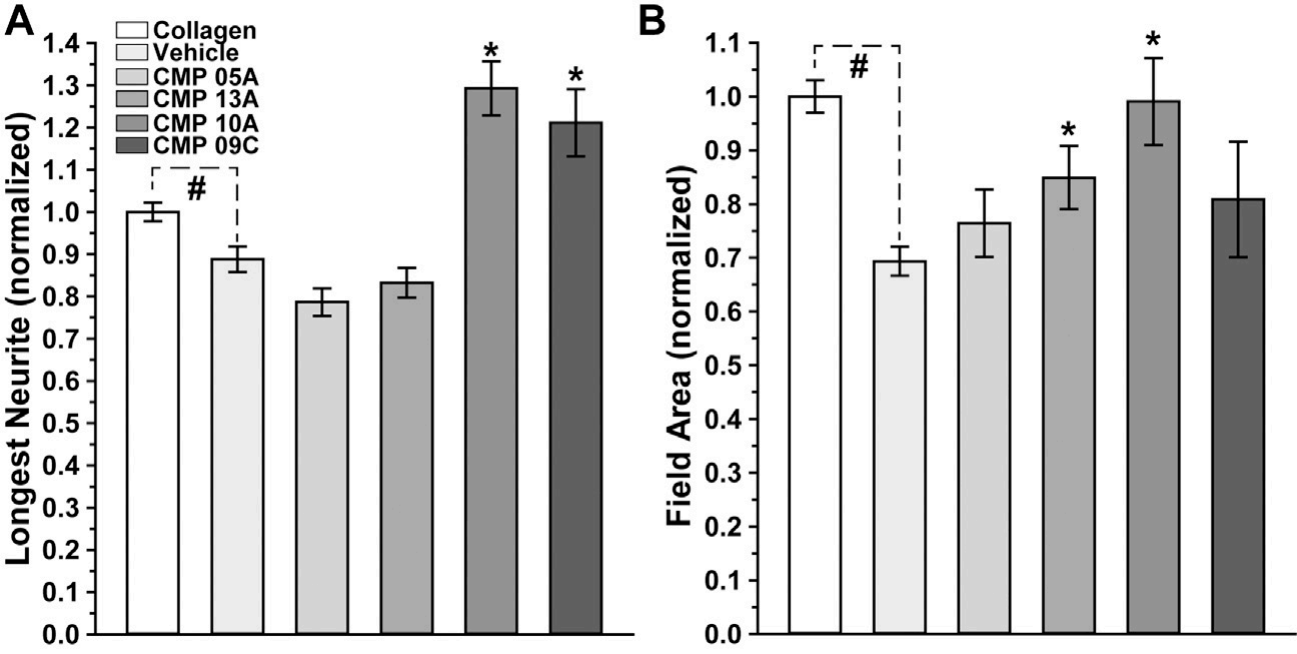
Neurite Outgrowth Following CMP Treatment. Longest neurite outgrowth **(A)** and explant field area **(B)** at 48 h normalized to their values for intact collagen (n = 178). Explants plated on digested collagen and treated with vehicle only (n = 139) demonstrate significantly diminished neurite length (*p* = 0.014) and field area (*p* < 0.001) compared to intact collagen (#). Treatment with CMPs 10A (n-17) and 09C (n = 16) extended the longest neurites, significantly beyond even intact collagen (*, *p* ≤ 0.036), while CMPs 13A (n = 17) and 10A increased field area compared to vehicle (*, *p* ≤ 0.03).

We quantified these trends by measuring both the longest neurite extending from each explant and the area of the field extending from and surrounding the explant with neurite coverage, both normalized to their values measured under conditions of intact collagen. Compared to intact collagen, the longest neurites of vehicle-treated explants plated on MMP-digested collagen were 15% shorter (*p* = 0.014; Figure 4A). In contrast, two CMPs of the four tested in this condition induced neurite outgrowth that was 29% (CMP 10A) and 21% (CMP 09C) greater than even intact collagen (*p* ≤ 0.036). Similarly, vehicle-treated explants demonstrated 30% smaller field areas compared to explants on intact collagen (*p* < 0.001; Figure 4B). Again, two CMPs significantly improved outgrowth compared to vehicle (*p* ≤ 0.03), one of which (CMP 10A) also induced greater neurite outgrowth.

### Collagen Mimetic Peptide Improves Axon Transport in Experimental Glaucoma

**FIGURE 5:**
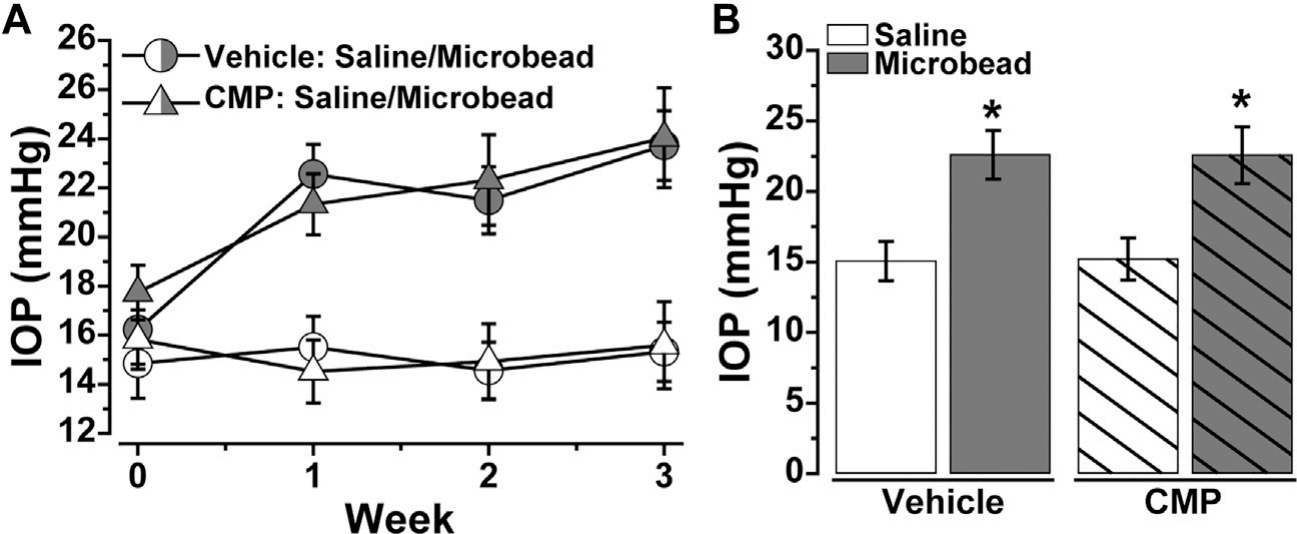
Ocular Delivery of a Mimetic Peptide Does Not Influence IOP. **(A)** Mean intraocular pressure (IOP) in mice treated with vehicle (○) vs. CMP 03A (Δ) prior to (day 0) and following (days ≥1) a single unilateral injection of polystyrene microbeads or equivalent volume of saline; CMP or vehicle delivery *via* intravitreal injection occurred on day 10 post-microbead. **(B)** Microbead injection significantly elevated IOP for both vehicle (22.59 ± 1.72 vs. 15.06 ± 1.40 mmHg, *p* < 0.001) and CMP (22.57 ± 2.01 vs. 15.21 ± 1.49 mm Hg, *p* < 0.001). IOP did not differ between cohorts for either the saline- or microbead-injected eye (*p* ≥ 0.70). Statistics: Student’s t-test (D). Data = mean ± SEM.

Our experiments with DRG explants demonstrate the reparative capacity of CMPs to promote neurite outgrowth under conditions of collagen degradation, at least *in vitro*. Recently we found that a similarly structured CMP promoted repair of corneal collagen and protection of epithelial cells *in vivo* (Baratta RO. et al., 2021). Here we used this same CMP (CMP 03A) to test whether it demonstrates a protective effect for neurons *in vivo*. To do so, we stressed the retinal ganglion cell (RGC) projection to the visual brain by elevating intraocular pressure (IOP) using microbead occlusion in mice (Sappington et al., 2010; Chen et al., 2011; Calkins et al., 2018). Prior to the procedure (day 0), baseline IOP for each eye did not differ between cohorts slated for vehicle vs. CMP treatment (*p* = 0.52; Figure 5A). Unilateral microbead injection induced a significant 40–50% elevation in IOP for both vehicle and CMP cohorts compared to the contralateral saline-injected eye that persisted through the 3-weeks experimental period (*p* < 0.001; Figure 5B). Ocular pressure did not differ between cohorts for either eye during this period (*p* ≥ 0.70).

Degradation of active anterograde axonal transport from retina to central brain targets is an early indicator of RGC degeneration in experimental glaucoma, both for chronic and acute models (Calkins et al., 2017; Cooper et al., 2020; Calkins, 2021). In mice with microbead-induced IOP elevations, transport deficits to the superior colliculus occur within 2–3 weeks, well before and as an antecedent to actual axonal degeneration in the optic nerve (Ward et al., 2014; Risner et al., 2018; Cooper et al., 2020). This is an important observation since the colliculus represents the primary target for RGCs in the rodent visual system. Furthermore, experimental interventions that slow or abate transport degradation also prevent subsequent stages of degeneration (Lambert et al., 2011, 2017, 2020; Dapper et al., 2013; Bernardo-Colón et al., 2018). With this in mind, we measured whether treatment with CMP could reduce the progression of anterograde transport degradation with IOP elevation.

**FIGURE 6:**
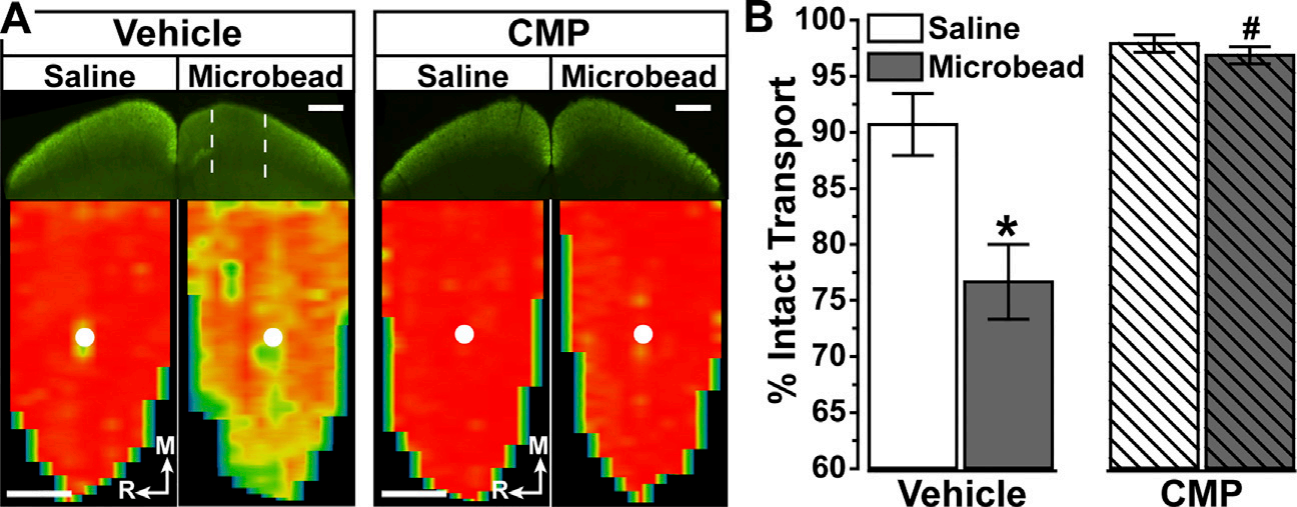
Collagen Mimetic Peptides May Preserve Anterograde Axon Transport. **(A)**. Single sections through superior colliculus **(top)** showing regions of intact CTB transport (green) for mice receiving either vehicle (n = 5) and CMP 03A (n = 4). Colliculus serving projection from the saline-injected control eye had fully intact transport, while microbead-induced IOP elevation in the fellow eye created regions of degraded transport (dotted lines) in vehicle-treated mice. Retinotopic maps **(bottom)** reconstructed from serial sections through colliculus with optic disc indicated (white circles). Levels of intact CTB signal ranges from 0% (blue) to 50% (green) to 100% (red). Medial (M) and rostral (R) orientations are indicated. Scale = 500 μm. **(B)**. For vehicle-treated mice, intact transport significantly declined with microbead-induced elevations in IOP compared to the colliculus from saline eye (*, *p* = 0.01). Compared to vehicle, CMP-treated microbead eyes demonstrated significant enhancement of transport to the colliculus (#, *p* = 0.015). There was no difference in transport between vehicle and CMP mice for saline controls (*p* = 0.44), and transport in drug treated mice showed no difference between microbead and saline eyes (*p* = 0.16). Data = mean ± SEM.

In vehicle-treated mice, 3 weeks of IOP elevation reduced RGC axonal transport from the retina to the colliculus, which is evident in the retinotopic spatial representation of intact CTB transport (Figure 6A). Intravitreal delivery of CMP appeared to protect against this degradation, as the retinotopic map of intact transport remained unchanged with elevated IOP compared to the control eye. When quantified, transport from vehicle-treated eyes with elevated IOP diminished by 15–20% (*p* = 0.01, Figure 6B), consistent with recent results (Risner et al., 2018; Cooper et al., 2020). In contrast, transport from CMP-treated eyes did not change with elevated IOP and was significantly protected compared to vehicle (*p* = 0.015).

## Discussion

Our fundamental finding is that certain mimetics of endogenous type I collagen have reparative or even protective properties for neurons under stress. In all tissues, collagen represents an important component of the ECM, and damage to collagen slows recovery from injury or disease (Sandhu et al., 2012). Under conditions of collagen challenged by MMP digestion (Figure 1), multiple CMPs promoted neurite outgrowth from dorsal root ganglia (DRG) explants, both in terms of longest neurite and area outside the explant represented by neurite coverage (Figures 2–4). These results are consistent with early studies showing that intact collagen is an important substrate for DRG growth (Sango et al., 1993; Hari et al., 2004), while delivery of ECM protein promotes neurite outgrowth following peripheral nerve injury (Deister et al., 2007). These results are even more impressive when we consider that DRG neurons in culture can release MMPs (Kawasaki et al., 2008), creating an additional challenge to CMP treatment.

Since DRG are a component of the peripheral nervous system (Berta et al., 2017), we also tested the *in vivo* efficacy of CMP treatment in protecting axonal function of retinal ganglion cells (RGCs), which form the optic projection of the central nervous system (CNS). With stress from elevated IOP, RGC axons undergo an early period of functional degradation prior to frank degeneration, marked by deficits in anterograde transport to central brain targets (Crish et al., 2010; Calkins, 2012; Crish and Calkins, 2015; Wareham et al., 2020). We showed that intraocular delivery of CMP during a period of elevated IOP ameliorated deficits in the retinotopic representation of active transport in the superior colliculus (Figures 5, 6), the primary target for RGC axons in rodents. Elevated IOP is an important risk factor for RGC degeneration and vision loss in glaucoma (Susanna et al., 2015), and slowing or stopping transport deficits in experimental models of glaucoma slows subsequent degenerative stages that occur in longer periods of microbead glaucoma, including axon degeneration followed by RGC body elimination (Lambert et al., 2011, 2017, 2020; Dapper et al., 2013). Interestingly, this same CMP (CMP 03A) promoted repair of the corneal epithelial layer following acute injury (Baratta RO. et al., 2021), thus motivating our use of it here.

In the adult CNS, ECM plays a critical role as a biologically active scaffold for maintaining biophysical stability and structure. Collagens, especially types I and IV, contribute to the matrix of the retina, optic nerve, and optic nerve head (Morrison et al., 1988; Sawaguchi et al., 1999; Huang et al., 2013). As in other tissues, collagen in these proximal structures of the optic projection is vulnerable to degradation and reorganization as matrix remodeling progresses in injury or disease. For example, following transection of monkey optic nerve, collagen type IV accumulates (Morrison et al., 1990), and levels of matrix metalloproteinases MMP3 and MMP9 increase along with tissue inhibitors of MMPs TIMP1 and TIMP2 (Agapova et al., 2003). Changes in the balance between MMP and TIMP levels are indicative of ongoing attempts at self-repair or remodeling, often with broad ramifications for axonal survival in disease or regeneration following acute trauma (Ahmed et al., 2005). In glaucoma, chronic sensitivity to IOP likely involves interplay between matrix degradation and synthesis of new matrix (Morrison et al., 1990; Wallace et al., 2014). Since similar changes in collagen content occur in optic nerve head and peripapillary sclera in glaucoma (Quigley et al., 1991), CMP treatment could ostensibly act in multiple ocular locations. Modulating MMP activity either pharmacologically or genetically reduces the chances for glial scarring and enhances the potential for RGC axon regeneration to preserve vision (Gaublomme et al., 2014; Tran et al., 2019).

We have argued earlier that a tissue repair technology that reestablishes collagen fibrillar organization could restore both biomechanical and structural functions of collagen membranes and normal and healthy cell signaling (Baratta R. O. et al., 2021), and ECM is increasingly viewed as an avenue for therapeutics (Ren et al., 2015). The results we describe here for DRG cultures and in our experimental glaucoma model support the idea that CMP treatment could accelerate repair or protect against further axonal injury through the established action of CMPs intercalating into damaged collagen strands (Chattopadhyay et al., 2012). This approach could represent a shift in how we address RGC axon degeneration. Experimental neuronal-based therapeutics for glaucoma or optic nerve trauma have relied nearly exclusively on targeting factors intrinsic to pro-apoptotic pathways, including oxidative, metabolic, inflammatory, and transcriptional stressors (Calkins, 2021). Because these pathways often involve redundant arms or are dependent on a delicate balance with other intracellular pathways, neuronal-based therapies have failed to reach the clinical ophthalmological market to any appreciable extent. Emerging preclinical approaches to retinal, optic nerve, and other CNS degeneration increasingly leverage delivery scaffolds comprising matrix-like or collagenous hydrogel (Xiong et al., 2009; Ren et al., 2015). Our approach differs fundamentally from these because it attempts not to use collagen to deliver a drug, but to directly repair endogenous collagen in the RGC axonal ECM to slow progression. The small number of CMPs tested here demonstrate modest reparative properties; we are examining whether other CMPs will show similar properties as we continue our studies with more extensive experimentation that incorporates additional outcome measures. A corollary is that this therapeutic approach could also reduce secondary inflammation that accelerates RGC degeneration in optic neuropathy that often arises with collagen degradation (Baratta R. O. et al., 2021). A simple intravitreal injection of CMP that could affect wide-spread repair of ECM and promote RGC survival could offer an alternative for millions suffering from optic neuropathies, both chronic and acute.

## Data Availability

The raw data supporting the conclusions of this article will be made available by the authors, without undue reservation.
